# OTEH-8. Pathway-based approach reveals sensitivity to radiation when targeting E2F1 in Glioblastoma

**DOI:** 10.1093/noajnl/vdab070.047

**Published:** 2021-07-05

**Authors:** Alvaro Alvarado, Kaleab Tessema, Sree Muthukrishnan, Riki Kawaguchi, Vivek Swarup, Steven Goldman, Harley Kornblum

**Affiliations:** 1 University of California, Los Angeles, Los Angeles, CA, USA; 2 University of California, Irvine, Irvine, CA, USA; 3 University of Rochester Medical Center, Rochester, NY, USA; 4 University of Copenhagen, Copenhagen, Denmark; 5 Jonsson Comprehensive Cancer Center, Los Angeles, CA, USA

## Abstract

The great phenotypic heterogeneity of glioblastoma (GBM) – both inter and intratumorally – has hindered therapeutic efforts. While genome-based molecular subtyping has revealed that GBMs may be parsed into several molecularly distinct categories, this insight has not translated to a significant extension of patient survival. We hypothesize that, rather than gene expression as a whole, analysis of targetable pathways could yield important insights into the development of novel classification schemes and, most importantly, to targeted therapeutics. Here, we interrogated tumor samples using a pathway-based approach to resolve tumoral heterogeneity. The Cancer Genome Atlas samples were clustered using gene set enrichment analysis and the resulting 3 clusters were informative of patient survival and only modestly overlapped with prior molecular classification. We validated our approach by generating gene lists from common elements found in the top contributing genesets for a particular cluster and testing the top targets in appropriate gliomasphere patient-derived lines. Samples enriched for cell cycle related genesets showed a decrease in sphere formation capacity, proliferation and in vivo tumor growth when E2F1, our top target, was silenced. Consistent with our theory, E2F1 knockdown had little or no effect on the growth of the non-enriched lines, despite their ability to proliferate in vitro and in vivo. We similarly analyzed single cell RNAseq datasets and correlated cell cycle and stemness signatures with the gene lists we generated, concluding that cells with stem cell signatures were depleted of E2F1 and its downstream targets. Finally, we confirmed a connection between E2F1 and cellular inhibitor of PP2A (CIP2A) in a cluster of samples. Loss of function studies reveal a diminished capacity for DNA damage regulation in E2F1 activated samples. Our studies relate inter- and intratumoral heterogeneity to critical cellular pathways dysregulated in GBM, with the ultimate goal of establishing a pipeline for patient- and tumor-specific precision medicine.

